# Publish and flourish, or the collective wisdom of peer review

**DOI:** 10.1098/rsnr.2022.0022

**Published:** 2022-09-20

**Authors:** Anna Marie Roos

**Affiliations:** *Editor-in-Chief*

When I think about peer review as Editor-in-Chief of *Notes and Records*, I remember to ask myself: why do scholars write? Is it curiosity, ambition, hope, courage or all those qualities blended into the creative impulse that says ‘I have some new knowledge, so what do *you* think?’

In the history of science, when editors select reviewers we hope that they will check that our potential contributor has read the historiography—in other words, that our author is aware of what other historians have said about the matter and sets her own argument in this scholarly context. We hope that our reviewers for *Notes and Records* are satisfied that the primary sources have been interpreted in a sensitive, nuanced and appropriate manner. Have the voices of the past been allowed to speak, or are they being shoehorned into an ill-fitting conceptual box for the sake of surface novelty and academic fashion? A case of Foucault for Foucault's sake?

We hope that our reviewers have considered whether the transcriptions of material are accurate. In the Hebrew Bible, when Moses came from Mount Sinai, his head was said to have ‘radiance’ or, in Hebrew, ‘karan’. However, when St Jerome was preparing his Latin edition of the Bible, he misread ‘karan’ as ‘keren’ or horned. Jerome's error meant that for generations, Moses was portrayed with horns, even by Michelangelo when sculpting Pope Julius II's tomb in 1513. Lost in translation.

We hope our reviewers check that authors understand the historical context of the materials with which they have chosen to work. Has the author recognized, for example, what the term ‘cousin’ in the eighteenth century really meant, or why seventeenth-century reflecting telescopes could perform poorly due to their speculum metal alloys? We hope that our reviewers offer suggestions on how to make the prose more fluid and compelling. Being a peer reviewer is demanding, and that is why it is especially important as an editor to use the principles of both diversity and expertise in selecting reviewers, and to check our *unconscious bias* all along the way. Voices from a variety of backgrounds and cultures allow us to assess a potential contribution to the field meaningfully. Unity in diversity.

And most of all, as editors, we have hopeful anticipation that the reviewers will say *yes*, this is new; *yes*, this is worth sharing; this is the creative impulse made manifest. Publication may take a while sometimes, as authors are finding their voice, or struggling with difficult material, or finding just the right words to make an event from the past come alive. But that's all right and to be expected, and publish or perish be damned. Let's remember that there is a deep breath taken before the author hits the ‘send’ button. Making knowledge is courageous.

As an editor, this process of refinement and care and scholarly exchange in the peer review process is a wonderful thing to observe and to facilitate. And the most wonderful result of successful peer review is to see a paper published, whether that is a young scholar making their debut in the scholarly community, or a more experienced colleague who tried something new and succeeded. If done correctly, both the peer reviewer and the author re-evaluate their expertise and communicate in a kind and collegial fashion—because, when it comes down to it, peer review in its ideal form is both an act of altruism and an act of investment in the continuation of the scholarly enterprise by our colleagues. That is why it is so important to engage in thoughtful peer review as a scholar, and that is why it is important to do it well, acting not as a gatekeeper, but as a fellow contributor in the creation of knowledge. Collective wisdom.

And I heartily thank all the peer reviewers for *Notes and Records* for their own collective wisdom and their excellent service to the journal.

